# lncRNA LUCAT1/ELAVL1/LIN28B/SOX2 Positive Feedback Loop Promotes Cell Stemness in Triple-Negative Breast Cancer

**DOI:** 10.1155/2022/7689718

**Published:** 2022-05-12

**Authors:** Li Xia, Hao Wang

**Affiliations:** Breast Surgery, Sichuan Cancer Hospital & Institute, Sichuan Cancer Center, School of Medicine, University of Electronic Science and Technology of China, Chengdu, Sichuan 610041, China

## Abstract

**Background:**

Triple-negative breast cancer (TNBC), as a subtype of breast cancer (BC), features an aggressive nature. Long noncoding RNAs (lncRNAs) are proved to get involved in the processes of cancers. lncRNA lung cancer associated transcript 1 (LUCAT1) has been reported in multiple cancers. The role of LUCAT1 in TNBC and its latent regulatory mechanism were investigated.

**Methods:**

RT-qPCR was performed to examine LUCAT1 expression. Functional experiments were implemented to disclose the role of LUCAT1 in TNBC. The underlying regulatory mechanism of LUCAT1 in TNBC was explored by chromatin immunoprecipitation (ChIP), RNA-binding protein immunoprecipitation (RIP), luciferase reporter, and RNA pull-down assays.

**Results:**

LUCAT1 is significantly overexpressed in TNBC cells. LUCAT1 interference impedes cell stemness in TNBC cells. SRY-box transcription factor 2 (SOX2) is an active transcription factor of LUCAT1. LUCAT1 recruits ELAV-like RNA binding protein 1 (ELAVL1) protein to stabilize lin-28 homolog B (LIN28B) mRNA, thereby further modulating SOX2 expression, which forms a positive feedback loop.

**Conclusion:**

The lncRNA LUCAT1/ELAVL1/LIN28B/SOX2 positive feedback loop promotes cell stemness in TNBC. The exploration of the mechanisms underlying TNBC stemness might be beneficial to TNBC treatment.

## 1. Introduction

Breast cancer (BC) remains a public-health issue all around the world, mainly threatening the health of women [1, 2]. As a subtype of BC, triple-negative breast cancer (TNBC) accounts for 15% to 20% of all BC cases [3]. Clinically, TNBC patients are characterized by greater invasiveness, a high rate of local recurrence, and organ metastasis [4]. Cancer stem cells (CSCs) have been reported to be the causes of chemoresistance, metastasis, and progression of TNBC [5]. The heterogeneity of TNBC leads to the complexity of TNBC therapy [6]. Nowadays, standardized TNBC treatment regiments are still limited and novel therapeutic strategies need to be explored [7]. Diminishing CSCs might help improve the efficacy of TNBC therapy [8].

Long non-coding RNAs (lncRNAs), as a class of noncoding RNAs, feature over 200 nucleotides in length and a lack of protein-coding potential. It is well recognized that lncRNAs occupy an important position in the progression of cancers [9], including TNBC [10]. For instance, Kong et al. have demonstrated that lncRNA PAPAS is an oncogene in TNBC via sequestering miR-34a [11]. Wang et al. have elucidated that lncRNA HCP5 promotes the malignant progression of TNBC by targeting the miR-219a-5p/BIRC3 axis [12]. Sun et al. have exposed that lncRNA TTN-AS1 influences TNBC development by interacting with miR-211-5p [13].

lncRNA lung cancer associated transcript 1 (LUCAT1) has been widely reported in diverse cancers. It has been proven that LUCAT1 propels the malignancy of colorectal cancer (CRC) by targeting the L40-MDM2-p53 pathway [14]. It has been demonstrated that LUCAT1 facilitates clear cell renal cell carcinoma (ccRCC) and cell proliferation and invasion via the AKT/GSK-3*β* pathway [15]. It has been verified that LUCAT1 promotes malignant progression of ovarian cancer (OvCa) by regulating the miR-612/HOXA13 pathway [16]. In addition, it has been validated that LUCAT1 serves as an oncogene in TNBC via binding to miR-5702 [17], and LUCAT1 is associated with a higher survival rate of TNBC patients [18]. However, the association of LUCAT1 with cell stemness in TNBC has never been reported before.

In this study, we focused on discussing the function of LUCAT1 on cell stemness in TNBC and probing into the underlying regulatory mechanism. Investigating the mechanisms underlying TNBC cell stemness is of great significance to TNBC therapy improvement.

## 2. Materials and Methods

### 2.1. Cell Culture

The TNBC cell lines (BT-549, MDA-MB-453, MDA-MB-231, and MDA-MB-468) and the human immortalized breast epithelial cell line (MCF10A) were all attained from the American Type Culture Collection (ATCC; Manassas, VA, USA). Among them, TNBC cell lines were all maintained in RPMI-1640 medium with 10% FBS (Gibco, NY, USA) while MCF-10A cell lines were in DMEM/F12 added with 100 ng/ml cholera toxin. All the cells were maintained in the mediums at 37°C with 5% CO_2_.

### 2.2. Cell Transfection

The short hairpin RNAs (shRNAs) targeting LUCAT1, SPIB, EZH2, FOXD3, LMNB1, PAX5, SOX2, ELAVL1, or LIN28B were designed by GenePharma (Shanghai, China). Furthermore, the sequences of SPIB, EZH2, FOXD3, LMNB1, PAX5, or SOX2 were subjected to insertion into pcDNA3.1 vectors to establish overexpression vectors. All transfections were implemented using Lipofectamine 2000 (Invitrogen, Carlsbad, CA, USA).

### 2.3. Quantitative Real-Time PCR (RT-qPCR) Analysis

Total RNA was isolated with TRIzol Reagent (Sigma, St. Louis, USA). Then, RNA was reverse transcribed into cDNA using M-MLV reverse transcriptase (Promega, Madison, WI, USA). Afterwards, GoTaq qPCR Master Mix (Promega) was adopted for PCR analysis. The relative expression was subjected to quantification based on 2^−ΔΔCt^ method. GAPDH or U6 were applied as the endogenous controls. The primers utilized in this study were supplemented as shown in Supplementary Table 1. The assay was repeated at least three times.

### 2.4. Bioinformatics Analyses

The GEO (https://www.ncbi.nlm.nih.gov/geo/) and HumanTFDB databases (http://bioinfo.life.hust.edu.cn/HumanTFDB#!/) were adopted to acquire the data of transcription factors of LUCAT1 under the conditions of Log2 fold change ≥3 and score ≥15, respectively. The starBase database (https://starbase.sysu.edu.cn/index.php) was utilized to screen the RNA-binding proteins (RBPs) of LUCAT1. The starBase and GEO databases were adopted to screen the downstream target mRNAs of LUCAT1 under the conditions of CLIP Data ≥5 and Log2 fold change ≥3. The JASPAR database (https://jaspar.genereg.net/) was applied to obtain the binding site sequence between SOX2 and the LUCAT1 promoter.

### 2.5. Spheroid-Formation Assay

TNBC cells were cultured in 24-well ultralow attachment plates (Corning, Shanghai, China) at 1000 cells/well by the use of the MammoCult™ Human Medium Kit (Cat#05620, Stemcell Technologies, Vancouver, BC, Canada). 10 days later, the cells were fixed and distinguished under the microscope. The eyepiece micrometer and stage micrometer were used to measure the size. The assay was repeated at least three times.

### 2.6. Flow Cytometry Assay

Oct4-positive cells were subjected to sorting via a flow cytometer. The top 2% of cells relative to the most brightly stained cells were chosen for the calculation of the proportion of Oct4-positive cells. The assay was repeated at least three times.

### 2.7. Subcellular Fractionation

Cytoplasmic and Nuclear RNA Purification Kit (NORGEN, Thorold, ON, Canada) was employed for this assay. GAPDH or U6 was utilized as the cytoplasmic or nuclear control, respectively. The assay was repeated at least three times.

### 2.8. Fluorescent In Situ Hybridization (FISH)

After being fixed in 4% PFA for 15 min at 37°C, TNBC cells were then subjected to permeabilization using 0.5% Triton X-100. Subsequently, the cells were hybridized with a LUCAT1-specific FISH probe in buffer, followed by counterstaining with Hoechst solution. Images were acquired using a confocal laser microscope (Olympus, Tokyo, Japan). The assay was repeated at least three times.

### 2.9. RNA Pull Down Assay

TNBC cells were lysed, followed by treatment with a biotin-labeled LUCAT1 probe. Next, a biotin-labeled RNA complex was added with magnetic beads. The beads were washed, and the RNAs in the pull-down products were isolated for the analysis of RT-qPCR. The assay was repeated at least three times.

### 2.10. RNA-Binding Protein Immunoprecipitation (RIP)

The RIP assay was carried out as per the instructions of the RNA-Binding Protein Immunoprecipitation Kit (Millipore, Burlington, MA, USA). Transfected TNBC cells were subjected to lysis in RIP buffer. Next, magnetic beads were conjugated with ELAVL1 (Abcam) antibody, LIN28B (Abcam) antibody or immunoglobulin G (IgG) (Abcam) antibody, followed by treatment with cell lysates. After the beads was washed, the RNA of the mixture were isolated for the detection of RT-qPCR. The assay was repeated at least three times.

### 2.11. Chromatin Immunoprecipitation (ChIP)

The EZ ChIP™ Chromatin Immunoprecipitation Kit (Millipore) was employed to implement the ChIP assay. In brief, the chromatin DNA was crosslinked, followed by sonication for chromatin fragment acquisition. Subsequently, the fragments were precipitated with SOX2 (Abcam, UK) antibody and IgG antibody (Abcam). Lastly, the immunoprecipitated DNA was analyzed through RT-qPCR. The assay was repeated at least three times.

### 2.12. Luciferase Reporter Assay

The LUCAT1 promoter region with the sequences of binding sites (wild type or mutant type) was inserted into the pGL3 vector (Promega, Madison, WI), along with the cotransfection of pcDNA3.1/SOX2 or the pcDNA3.1 vector into TNBC cells. 48 h later, luciferase activity was subjected to analysis by the Dual-Luciferase Reporter Gene Assay Kit (Beyotime, Shanghai, China). The assay was repeated at least three times.

### 2.13. Western Blot Analysis

Total proteins isolated from cells were treated with SDS-PAGE for separation, followed by being transferred onto PVDF membranes (Thermo Fisher, IL, USA). Next, membranes were blocked in defatted milk, followed by incubation with primary antibodies overnight at 4°C. The blots were incubated with secondary antibody for 1 h in a dark room subsequent to the washes in PBS three times. The antibodies against Oct4, Nanog, SOX2, ELAVL1, LIN28B, and *β*-actin were commercially attained from Abcam. The protein was quantified by the chemiluminescence system (GE Healthcare, Chicago, USA). The assay was repeated at least three times.

### 2.14. Statistical Analysis

The experimental data presented in the study exhibited as the mean ± standard deviation (SD), with SPSS 22.0 statistical software as tool for data analysis. Student's *t*-test or analysis of variance (ANOVA) was employed to analyze significant differences between two or more groups. All the assays were independently carried out in triplicate. *P* value lower than 0.05 was regarded as the criterion for statistical significance.

## 3. Results

### 3.1. lncRNA LUCAT1 Is Highly Expressed in TNBC Cells

**Figure 1:**
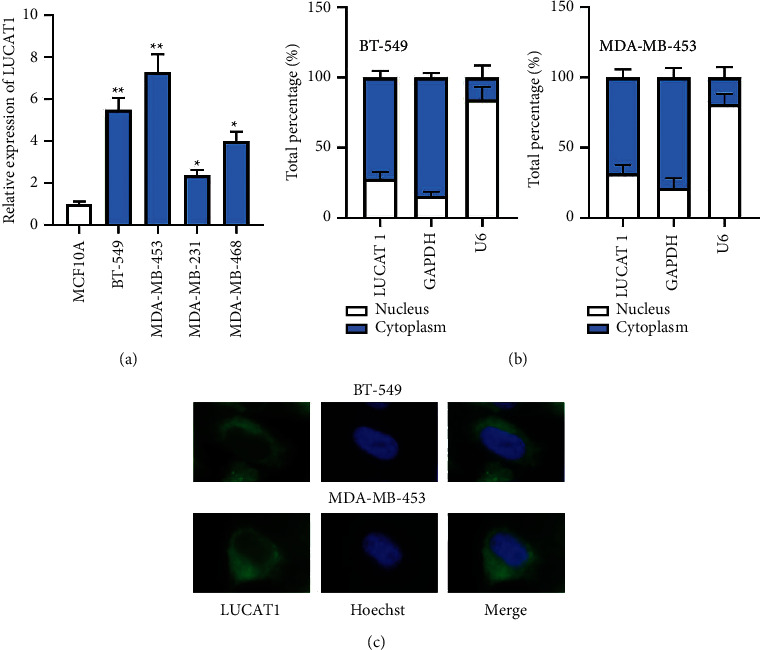
lncRNA LUCAT1 is highly expressed in TNBC. (a) RT-qPCR examined LUCAT1 expression in TNBC cell lines and normal cell line. (b, c) The accumulation of LUCAT1 in TNBC cells was investigated via subcellular fractionation and FISH assays.  ^*∗*^*P* < 0.05,  ^*∗∗*^*P* < 0.01.

Firstly, RT-qPCR was utilized to examine LUCAT1 expression and the result indicated that LUCAT1 was upregulated in TNBC cell lines (BT-549, MDA-MB-453, MDA-MB-231, and MDA-MB-468) in comparison with normal cell lines (MCF10A) (Figure 1(a)). We selected BT-549 and MDA-MB-453 cells for follow-up assays due to higher expression of LUCAT1. Besides, subcellular fractionation and FISH assays were implemented to explore the distribution of LUCAT1 in TNBC cells. It was shown that LUCAT1 was mainly accumulated in the cytoplasm of TNBC cells (Figures 1(b) and 1(c)).

### 3.2. Depletion of LUCAT1 Impedes Cell Stemness in TNBC

**Figure 2:**
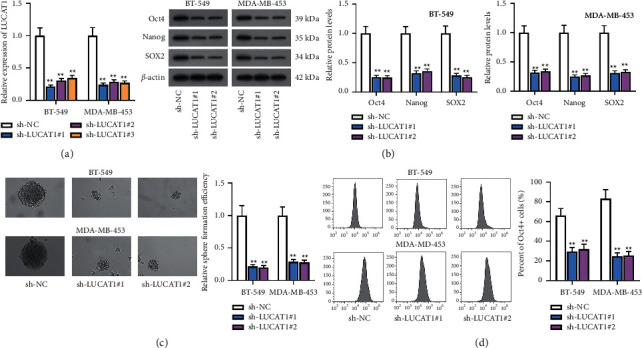
Depletion of LUCAT1 impedes cell stemness in TNBC. (a) LUCAT1 expression was evaluated by RT-qPCR in BT-549 and MDA-MB-453 cells after the transfection of sh-LUCAT1#1/2/3. (b) Western blot analyzed Oct4, Nanog, and SOX2 protein levels when LUCAT1 expression was downregulated. (c) Spheroid-formation assay was implemented to observe spheroids after LUCAT1 knockdown. (d) Flow cytometry assay detected the percentage of Oct4-positive cells after LUCAT1 knockdown.  ^*∗∗*^*P* < 0.01.

To assess the role of LUCAT1 in TNBC, sh-LUCAT1#1/2/3 plasmids were subjected to transfection into BT-549 and MDA-MB-453 cells to knock down LUCAT1 (Figure 2(a)). A western blot was implemented to evaluate the protein levels of CSC transcription factors (Oct4, Nanog, and SOX2). The results disclosed that when LUCAT1 expression was downregulated, Oct4, Nanog, and SOX2 protein levels were all decreased (Figure 2(b)). Meanwhile, spheroid-formation and flow cytometry assays disclosed that cell stemness was inhibited due to LUCAT1 silencing (Figures 2(c) and 2(d)).

### 3.3. SOX2 Is a Transcription Activator of LUCAT1

**Figure 3:**
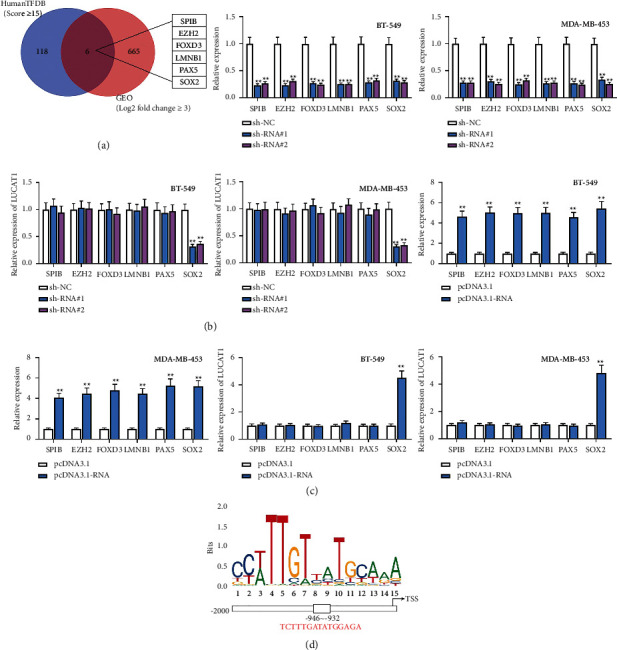
SOX2 is a transcription activator of LUCAT1. (a) Venn diagram demonstrated 6 potential transcription factors. (b) RT-qPCR analysis detected the expression of 6 transcription factors and LUCAT1 when the expression levels of these 6 transcription factors were respectively impeded. (c) RT-qPCR analysis evaluated the expression of 6 transcription factors and LUCAT1 when these 6 transcription factors were respectively overexpressed. (d) JASPAR database predicted the motif of SOX2 and binding sites between SOX2 and LUCAT1 promoter.  ^*∗∗*^*P* < 0.01.

Subsequently, the upstream mechanism of LUCAT1 in TNBC was investigated. Based on GEO and HumanTFDB databases, the Venn diagram exhibited 6 potential transcription factors (SPIB, EZH2, FOXD3, LMNB1, PAX5, and SOX2) (Figure 3(a)). The knockdown efficiency of sh-SPIB#1/2, sh-EZH2#1/2, sh-FOXD3#1/2, sh-LMNB1#1/2, sh-PAX5#1/2, and sh-SOX2#1/2 was assessed by RT-qPCR. Furthermore, we detected LUCAT1 expression after the transfection of these vectors and observed that LUCAT1 expression was reduced when SOX2 was inhibited (Figure 3(b)). We then performed RT-qPCR to detect overexpression efficiency of pcDNA3.1-SPIB, pcDNA3.1-EZH2, pcDNA3.1-FOXD3, pcDNA3.1-LMNB1, pcDNA3.1-PAX5, and pcDNA3.1-SOX2. Moreover, LUCAT1 expression was detected after the transfection of the overexpression vectors. It was shown that, when SOX2 was overexpressed, LUCAT1 expression was accordingly enhanced (Figure 3(c)). Hence, SOX2 was selected for subsequent experiments. The binding sequence between SOX2 and LUCAT1 promoter was predicted by JASPAR database as exhibited in Figure 3(d). ChIP assay verified that SOX2 had a strong affinity with the LUCAT1 promoter (Figures S1A). In addition, the luciferase reporter assay showed that upregulated SOX2 expression level caused an evident increase in the luciferase activity of the wild type group, namely, the LUCAT1 promoter-Wt group (Figure S1B).

### 3.4. LUCAT1 Interacts with ELAVL1 Protein

**Figure 4:**
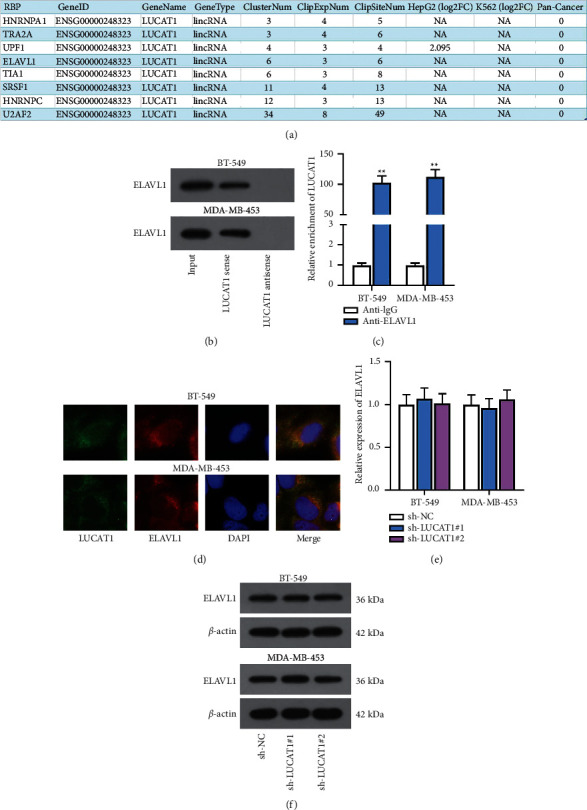
LUCAT1 interacts with ELAVL1 protein. (a) starBase predicted potential RBPs. (b) The interaction between LUCAT1 and ELAVL1 was verified by RNA pull down assay. (c) RIP assay testified the binding between ELAVL1 and LUCAT1. (d) FISH and IF analysis displayed the colocalization of LUCAT1 and ELAVL1 in TNBC cells. (e, f) ELAVL1 expression was tested when LUCAT1 was silenced.  ^*∗∗*^*P* < 0.01.

As shown in the previous results, LUCAT1 was prominently located in the cytoplasm of TNBC cells. Hence, we speculated that LUCAT1 might interact with certain proteins at posttranscriptional levels. As demonstrated in Figure 4(a), starBase predicted 8 potential RBPs (HNRNPA1, TRA2A, UPF1, ELAVL1, TIAL, SRSF1, HNRNPC, and U2AF2). Among them, ELAVL1 was chosen due to the fact that it is a common RBP in human diseases. The RNA pull down assay indicated that LUCAT1 has a strong affinity with ELAVL1 as evidenced by the enrichment of ELAVL1 in the LUCAT1 sense group (Figure 4(b)). Moreover, the RIP assay displayed the enrichment of LUCAT1 in the ELAVL1 antibody, indicating the interaction between LUCAT1 and ELAVL1 (Figure 4(c)). The colocalization of LUCAT1 and ELAVL1 in the cytoplasm of TNBC cells was demonstrated by FISH and IF analysis (Figure 4(d)). According to RT-qPCR and western blot analysis, we noticed that LUCAT1 downregulation exerted no influence on ELAVL1 expression, indicating that LUCAT1 cannot directly regulate ELAVL1 expression (Figures 4(e) and 4(f)).

### 3.5. LUCAT1 Stabilizes LIN28B mRNA by Binding to ELAVL1

**Figure 5:**
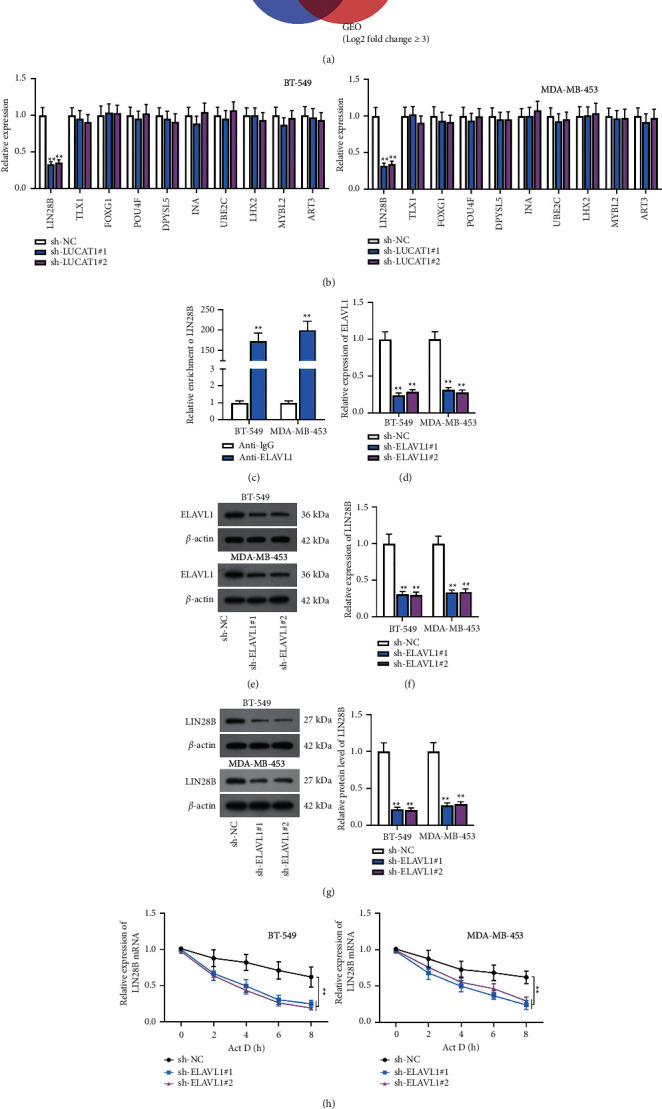
LUCAT1 stabilizes LIN28B mRNA by binding to ELAVL1. (a) Venn diagram showed 10 upregulated mRNAs which could also bind to ELAVL1. (b) The expression of 10 mRNAs was detected after LUCAT1 was inhibited. (c) The affinity of ELAVL1 and LIN28B was determined by RIP assay. (d, e) ELAVL1 expression was reduced in BT-549 and MDA-MB-453 cells. (f, g) The mRNA levels and protein levels of LIN28B were examined when ELAVL1 was downregulated. (h) LIN28B mRNA stability was tested after ELAVL1 was depleted.  ^*∗∗*^*P* < 0.01.

Through the starBase and GEO databases, 10 mRNAs were predicted as shown in Venn diagram (Figure 5(a)). RT-qPCR analyzed that only LIN28B expression was decreased after LUCAT1 knockdown (Figure 5(b)). The interaction between ELAVL1 and LIN28B was also validated by the RIP assay (Figure 5(c)). Afterwards, the efficiency of sh-ELAVL1#1/2 was detected by RT-qPCR and western blot. It was shown that ELAVL1 expression was downregulated in BT-549 and MDA-MB-453 cells (Figures 5(d) and 5(e)). Subsequently, we performed RT-qPCR and western blot, finding that LIN28B expression was decreased after ELAVL1 silencing (Figures 5(f) and 5(g)). Furthermore, it was found that LIN28B mRNA level was declined after ELAVL1 knockdown in TNBC cells treated with actinomycin D (Act D), indicating that LIN28B stability was evidently reduced due to ELAVL1 deficiency (Figure 5(h)).

### 3.6. LIN28B Modulates SOX2 Expression by Stabilizing SOX2 mRNA

**Figure 6:**
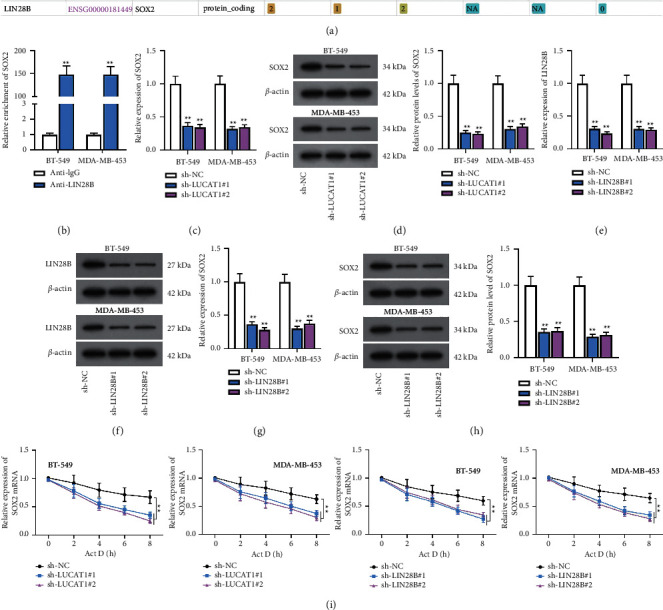
LIN28B modulates SOX2 expression by stabilizing SOX2 mRNA. (a) starBase predicted the potential binding between LIN28B and SOX2. (b) RIP assay disclosed the interaction between LIN28B and SOX2. (c, d) RT-qPCR and western blot analyzed SOX2 expression after LUCAT1 was knocked down. (e, f) LIN28B expression was decreased in BT-549 and MDA-MB-453 cells. (g, h) SOX2 expression was detected when LIN28B was silenced. (i) SOX2 mRNA stability was tested when LUCAT1 or LIN28B was downregulated.  ^*∗∗*^*P* < 0.01.

We predicted that SOX2 is also a target gene of LIN28B through starBase (Figure 6(a)). Therefore, we speculated that the LUCAT1/ELAVL1/LIN28B/SOX2 axis forms a positive feedback loop in TNBC cells. The experimental results of RIP assay displayed the relationship between LIN28B and SOX2 (Figure 6(b)). Additionally, it was unearthed by RT-qPCR and western blot that SOX2 mRNA and protein levels were both decreased after LUCAT1 was downregulated (Figures 6(c) and 6(d)). The efficiency of sh-LIN28B#1/2 was detected by RT-qPCR and western blot (Figures 6(e) and 6(f)). SOX2 expression was also inhibited due to silencing of LIN28B (Figures 6(g) and 6(h)). Eventually, as demonstrated by the results of RT-qPCR, the stability of SOX2 mRNA was weakened after LUCAT1 or LIN28B was knocked down (Figure 6(i)).

### 3.7. LUCAT1 Contributes to Cell Stemness in TNBC via Enhancing SOX2 Expression

**Figure 7:**
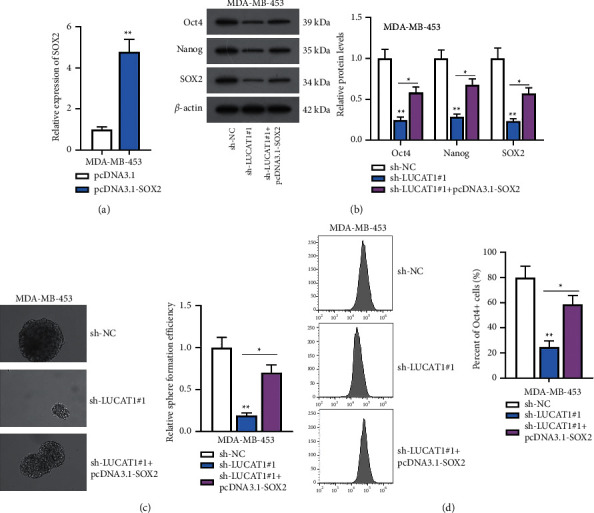
LUCAT1 contributes to cell stemness in TNBC via enhancing SOX2 expression. (a) SOX2 was overexpressed in MDA-MB-453 cells. (b) Oct4, Nanog, and SOX2 protein levels were analyzed in the sh-NC group, sh-LUCAT1#1 group, and sh-LUCAT1#1+pcDNA3.1-SOX2 group. (c) Cell stemness was observed via spheroid-formation assay in the sh-NC group, sh-LUCAT1#1 group, and sh-LUCAT1#1+pcDNA3.1-SOX2 group. (d) Flow cytometry assay detected cell stemness in the sh-NC group, sh-LUCAT1#1 group, and sh-LUCAT1#1+pcDNA3.1-SOX2 group.  ^*∗*^*P* < 0.05,  ^*∗∗*^*P* < 0.01.

Before the implementation of rescue assays, the efficiency of pcDNA3.1-SOX2 was overexpressed in MDA-MB-453 cells (Figure 7(a)). Western blot analysis uncovered that LUCAT1 depletion led to the downregulation of Oct4, Nanog, and SOX2 protein levels, while this effect was rescued by SOX2 overexpression (Figure 7(b)). Likewise, the experimental results of spheroid-formation and flow cytometry assays manifested that SOX2 upregulation restored the suppressed cell stemness caused by LUCAT1 deficiency (Figures 7(c) and 7(d)).

## 4. Discussion

Accumulating evidence has discovered that lncRNAs widely participate in the tumorigenesis and metastasis of cancers [9]. As exhibited in the current study, lncRNA LUCAT1 was found to be overexpressed in TNBC cells, suggesting its oncogenic role in TNBC. On the basis of a literature review, LUCAT1 has been found to play an oncogenic role in diverse cancers, including TNBC. For instance, Chi et al. have disclosed that lncRNA LUCAT1 stimulates the progression of gastric cancer via ceRNA mode [19]. Yu et al. have attested that LUCAT1 plays a promoting role in ovarian cancer via sponging miR-612 and regulating HOXA13 expression [16]. Nai et al. have certified that LUCAT1 influences the proliferation and migration of pancreatic ductal adenocarcinoma cells through interacting with miR-539 [20]. As for TNBC, it has previously been proven that LUCAT1 induces TNBC tumorigenesis and metastasis via binding to miR-5702 [17]. In our study, we deeply investigated the effects of LUCAT1 on the stemness of TNBC cells. Through performing several experiments, we discovered that silencing of LUCAT1 led to the inhibition of cell stemness in TNBC, indicating that LUCAT1 facilitates TNBC cell stemness.

As a common transcription factor, SOX2 has a high affinity with lncRNAs [21]. Moreover, SOX2 is implicated in the metastasis and tumorigenesis of cancers [22]. Our experimental results manifested that SOX2 interacts with the promoter of LUCAT1 and induces the upregulation of LUCAT1 in TNBC cells.

RBPs have been considered to be crucial regulators in posttranscriptional events [23]. In accordance with the results of subcellular fractionation and FISH assays, LUCAT1 was found to be prominently located in the cytoplasm of TNBC cells which indicated that LUCAT1 might function at a posttranscriptional level. ELAVL1, or Hu-antigen R (HUR), is capable to stabilize mRNA and is of great significance in human malignant cancers [24]. Similarly, we concluded that ELAVL1 is recruited by LUCAT1 in TNBC cells, while its expression cannot be influenced by LUCAT1 depletion.

LIN28B has been verified to be an active participator in BC [25]. Meanwhile, it can also serve as an RBP to regulate the stability of mRNAs. Our investigation uncovered that LUCAT1 stabilizes LIN28B mRNA by binding to ELAVL1. In addition, it has been concluded that LIN28B can also stabilize SOX2 mRNA to modulate SOX2 expression. The results of rescue assays demonstrated that SOX2 overexpression rescued the suppressive role of LUCAT1 silencing in TNBC cell stemness. To conclude, the LUCAT1/ELAVL1/LIN28B/SOX2 positive feedback loop is formed in TNBC.

## 5. Conclusion

Collectively, LUCAT1 is overexpressed in TNBC cells, and inhibition of LUCAT1 depresses cell stemness in TNBC. LUCAT1 mediated by SOX2 interacts with ELAVL1 protein and stabilizes LIN28B expression, thereby influencing cell stemness in TNBC through modulating SOX2 expression in turn. All these findings in the current study contributed to the understanding of the mechanisms underlying TNBC cell stemness, which is of significance to TNBC treatment development. However, our study can be improved in some aspects. Although we have validated the role of LUCAT1 in TNBC cell stemness, animal experiments are still required to investigate the mechanisms in vivo. Furthermore, clinicopathological analyses remain to be conducted. In the future, we will explore the tumorigenesis and clinicopathological relevance of LUCAT1 to deepen our understanding of the mechanisms underlying cell stemness.

## Data Availability

The datasets generated during or analyzed during the current study are available from the corresponding author on reasonable request.

## Supplementary Materials

Supplementary Materials Figure S1A ChIP assay was conducted to examine the interaction between LUCAT1 promoter and SOX2. (B) Luciferase reporter assay validated the binding between SOX2 and LUCAT1 promoter.  ^*∗∗*^*P* < 0.01. Supplementary Table 1. The sequences of primers.
