# Supplementary material for: lncRNA LUCAT1/ELAVL1/LIN28B/SOX2 Positive Feedback Loop Promotes Cell Stemness in Triple-Negative Breast Cancer
Source: Breast J. 2022 May 12;2022:7689718. doi: 10.1155/2022/7689718 (PMC9187271; doi:10.1155/2022/7689718)
Supplement: Supplementary Materials — Figure S1A ChIP assay was conducted to examine the interaction between LUCAT1 promoter and SOX2. (B) Luciferase reporter assay validated the binding between SOX2 and LUCAT1 promoter. ∗∗P < 0.01. Supplementary Table 1. The sequences of primers. [file 7689718.f1.zip › 7689718.f1/Supplementary Table 1.docx]

| The sequences of primers | |
| --- | --- |
| gene | primer |
| LUCAT1 | F: CCGGCTTTATTGTCGCTTCG R: CACACCGATCCTCCACACAG |
| SPIB | F: CCTATGAAGCCTTCGACCCG R: GGCTGTCCAACGGTAAGTCT |
| EZH2 | F: GGACTCAGAAGGCAGTGGAG R: CTTGAGCTGTCTCAGTCGCA |
| FOXD3 | F: GCAACTACTGGACCCTGGAC R: CTGTAAGCGCCGAAGCTCT |
| LMNB1 | F: CAGTCACCCTCGTCTTGCAT R: GGGAGCTCGATTACAGCACA |
| PAX5 | F: TCCGCCAGAGGATAGTGGAA R: ATGGAACTGACGCTAGGCAC |
| SOX2 | F: AACCAGCGCATGGACAGTTA R: GACTTGACCACCGAACCCAT |
| ELAVL1 | F: GGGAGGCTCTCTTAACCGAT R: GCTGATGGAAAACTGATAAGGGC |
| LIN28B | F:GCACATTAGACCATGCGAGC R:ACTTACAGTGGCCAGTTCCG |
| TLX1 | F: AGGTTCACAGGTCACCCCTA R: AGTCTGCCGTCTCCACTTTG |
| FOXG1 | F: TGCCAAGTTTTACGACGGGA R: AGTCTGGTCCCAGGGATGTT |
| POU4F | F: GTGGATATACATGCCAAGCCG R: CGGCTTGAAAGGATGGCTCT |
| DPYSL5 | F: CATCTCGGAGGAGTGGGTGATG R: GCGTACAAAGAGGACCCCAAG |
| INA | F: GAGATCCACGAGTATCGGCG R: AAAACGTGTCTCCTCGCCTT |
| UBE2C | F: CGAGTTCCTGTCTCTCTGCC R: TGCTCCATGGATGGTCCCTA |
| LHX2 | F: CCAACTGTGACGTCCGTCTT R: AGTTGTTCCTCGGTCCACAC |
| MYBL2 | F: GTGAGGCAGTTTGGACAGCA R: CTCAGGGTTGAGGTGGTTGT |
| ART3 | F: GAACCCACCCAAATACCTGGA R: GGAACAGGAACTGGACCTGG |

F: forward primer R: reverse primer
