# Supplementary figures and images for: lncRNA LUCAT1/ELAVL1/LIN28B/SOX2 Positive Feedback Loop Promotes Cell Stemness in Triple-Negative Breast Cancer
Source: Breast J. 2022 May 12;2022:7689718. doi: 10.1155/2022/7689718 (PMC9187271; doi:10.1155/2022/7689718)

**A**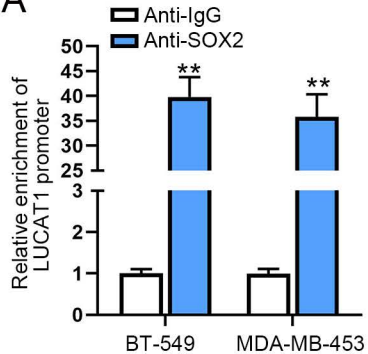**B**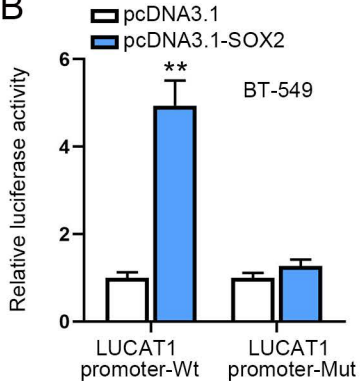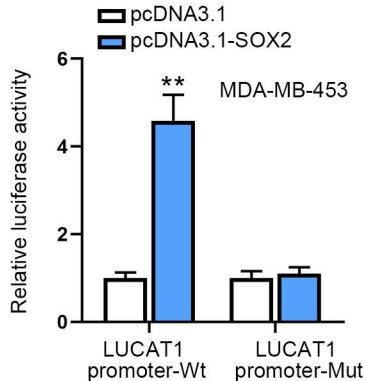

Supplement: Supplementary Materials — Figure S1A ChIP assay was conducted to examine the interaction between LUCAT1 promoter and SOX2. (B) Luciferase reporter assay validated the binding between SOX2 and LUCAT1 promoter. ∗∗P < 0.01. Supplementary Table 1. The sequences of primers. [file 7689718.f1.zip › 7689718.f1/Figure S1.pdf]
